# Reply to Klevay, L.M. Comment on “Huang et al. Influence of Varied Dietary Cholesterol Levels on Lipid Metabolism in Hamsters. *Nutrients* 2024, *16*, 2472”

**DOI:** 10.3390/nu17121945

**Published:** 2025-06-06

**Authors:** Chung-Hsiung Huang, Hung-Sheng Hsu, Meng-Tsan Chiang

**Affiliations:** Department of Food Science, College of Life Sciences, National Taiwan Ocean University, Keelung 20224, Taiwan

Thank you for your insightful comments and suggestions [1]. We agree with your point that copper deficiency induced by cholesterol feeding has been well established in previous studies [2,3]. Our findings of decreased hepatic glutathione levels, which resemble those observed in copper-deficient models [4], highlight the potential role of oxidative stress in cholesterol-induced cardiovascular processes.

Regarding the observed decrease in lipid peroxidation despite the apparent copper deficiency, we acknowledge the complexity of this relationship. Variability in the effects of copper deficiency on lipid peroxidation has been noted, with reports of a wide range of metabolic changes [5]. This variability may suggest that lipid peroxidation is comparatively resistant to copper deficiency in certain contexts, which warrants further investigation.

We appreciate your suggestion to include a dietary copper supplementation group in our experiments. We agree that measuring copper status via markers such as serum copper, ceruloplasmin, and superoxide dismutase (SOD) would provide valuable insights into the role of copper in our observed changes. As noted, SOD, in particular, is an important antioxidant enzyme that may play a critical role in mitigating oxidative damage [6]. We will consider incorporating these measurements in future studies to better understand how copper status influences the outcomes in our cholesterol-fed hamster model.

Thank you again for your helpful feedback, which will inform our future research direction.
